# Ensuring Sufficient Trough Plasma Concentrations for Broad-Spectrum Beta-Lactam Antibiotics in Children With Malignancies: Beware of Augmented Renal Clearance!

**DOI:** 10.3389/fped.2021.768438

**Published:** 2022-01-05

**Authors:** Pascal André, Léonore Diezi, Kim Dao, Pierre Alex Crisinel, Laura E. Rothuizen, Haithem Chtioui, Laurent Arthur Decosterd, Manuel Diezi, Sandra Asner, Thierry Buclin

**Affiliations:** ^1^Service of Clinical Pharmacology, University Hospital Center, University of Lausanne, Lausanne, Switzerland; ^2^Pediatric Infectious Diseases and Vaccinology Unit, Service of Pediatrics, University Hospital Center, University of Lausanne, Lausanne, Switzerland; ^3^Pediatric Hemato-Oncology Unit, Service of Pediatrics, University Hospital Center, University of Lausanne, Lausanne, Switzerland

**Keywords:** meropenem, piperacillin, beta-lactams, child, neoplasms, cancer, kidney function tests, augmented renal clearance

## Abstract

**Introduction:** Broad-spectrum beta-lactams are commonly prescribed for empirical or selective treatment of bacterial infections in children with malignancies. In the immunocompromised, appropriate concentration exposure is crucial to ensure antimicrobial efficacy. Augmented renal clearance (ARC) is increasingly recognized in this population, and raises concern for unmet concentration targets. We conducted a retrospective evaluation of meropenem and piperacillin exposure in our hospital's pediatric hematology-oncology patients.

**Materials and Methods:** We compared trough levels of meropenem and piperacillin in a cohort of unselected pediatric hematology-oncology patients stratified based on their estimated renal function as decreased, normal or with ARC, and on their neutrophil count.

**Results:** Thirty-two children provided a total of 51 meropenem and 76 piperacillin samples. On standard intermittent intravenous regimen, 67% of all trough plasma concentrations were below targeted concentrations. In neutropenic children with bacterial infection, all meropenem and 60% of piperacillin levels were below target. Nearly two-thirds of total samples came from children with ARC. In these patients, antimicrobial exposure was insufficient in 85% of cases (compared to 36% in the decreased or normal renal function groups), despite a dosage sometimes exceeding the maximum recommended daily dose. Under continuous infusion of piperacillin, only 8% of plasma levels were insufficient.

**Discussion:** Intermittent administration of meropenem and piperacillin often fails to ensure sufficient concentration exposure in children treated for malignancies, even at maximal recommended daily dosage. This can in part be attributed to ARC. We recommend thorough assessment of renal function, resolute dosage adjustment, continuous infusion whenever possible and systematic therapeutic drug monitoring.

## Introduction

In high-income countries, the survival rate of children affected by malignancies is constantly improving, currently reaching between 80% to nearly 90% 5 years overall survival, primarily thanks to advances in anticancer treatment efficacy and supportive care (1, 2). Yet malignancies remain a leading cause of death during childhood and adolescence in these countries (3, 4). A significant part of this mortality results from their susceptibility to develop bacterial, fungal, and viral systemic infections as a result of secondary neutropenia and immunodeficiency. Mortality due to infections depends on the type of cancer, but it is considered highest for hematological malignancies, mainly acute lymphoblastic leukemia (ALL) and acute myeloid leukemia (AML), which carry a greater burden of disease or treatment-related myelosuppression. In contemporary ALL trials, the frequency of treatment-related mortality is reported to be between 2 and 4%, primarily due to bacterial infections (5–8). In lymphoma, mortality attributed to infection is also significant (9). Thus, bacterial infections are a definite threat for children with malignancies, with leukemia and lymphoma being the most frequent types of cancer occurring between ages 0 to 14 years and 15 to 19 years, respectively (10). Bacterial infections also complicate solid tumors treatments, although to a lesser extent (11, 12).

In neutropenic episodes that follow ALL treatment, infection-related lethality most often occurs within 48 h from first clinical signs of infection, emphasizing the need for prompt diagnosis and effective antibiotic treatment (7). Therefore, the antibiotic should typically be initiated within an hour from any developing sign of sepsis in an immunocompromised patient. The choice is empirical as treatment is initiated before identification of the infectious agent involved, and frequently contains a broad-spectrum beta-lactam. Considering the time-dependent action of beta-lactams, it is widely recommended to maintain free trough blood concentrations above the minimal inhibitory concentration (MIC) of common germs in these patients (13, 14). Indeed the unbound fraction of beta-lactams, which varies between molecules, is sole responsible for the antibacterial effect (15). Because intensive therapy is required, special attention is also to be paid to potential toxicity of beta-lactams, mainly presenting as adverse neurological effects. These antibiotics are predominantly eliminated by renal excretion, through glomerular filtration and active tubular secretion, and recommendations to reduce the unit dose or prolong the dose interval depending on the stages of renal failure is generally acknowledged (16). Accordingly, maximum plasma levels recommended at trough have been formally defined for some beta-lactams (17).

Recently, the concept of augmented renal clearance (ARC), also termed glomerular hyperfiltration, has emerged in clinical medicine. It is defined as a global enhancement of renal function, typically observed in the critically ill, which accelerates clearance of drugs excreted by the kidney (18). It is thought to result from recruiting renal functional reserve in response to inflammation, systemic vasodilation with hyperdynamic circulation, high cardiac output, increased splanchnic blood flow and a global raise in metabolic activity (19). It is characterized by an increase in creatinine clearance above a threshold not clearly agreed upon to date, which varies between 130 mL/min/1.73 m^2^ (20) and 170 mL/min/1.73 m^2^ (21) according to different authors for adult patients. Overall, ARC is a multiprocess phenomenon comprising enhanced glomerular filtration but presumed to modulate tubular transport as well, notably tubular anionic secretion and reabsorption. This wider term is preferred to glomerular hyperfiltration, which refers only to the enhanced filtration component (22, 23). While initially described in adult patients experiencing acute inflammatory conditions (21), ARC seems no less observed in the pediatric population, with episodes of ARC reported in 30 to 40% of children with malignancies. These episodes are most prevalent before starting and just after the first course of chemotherapy (23), and become less frequent with increasing courses of chemotherapy. Mechanisms hypothesized to contribute to ARC in these observations relate to the hypermetabolic state resulting from the multiplication of malignant cells prior to treatment, and the tumor cells lysis after chemotherapy (23). ARC has been shown to reduce drug exposure, in particular to antibiotics eliminated by glomerular filtration with or without tubular secretion.

The broad-spectrum beta-lactams most frequently used to for the empirical treatment of febrile neutropenia are meropenem (MER) and piperacillin (PIP), which belong to the carbapenem and the ureidopenicillin families, respectively. PIP is combined with the beta-lactamase inhibitor tazobactam. In order to adapt their dosage to a specific clinical state, precision medicine resorts to therapeutic drug monitoring (TDM). This requires to draw and measure plasma concentrations of the drug, and eventually adapt the dosage according to the result, in order to prevent treatment failure due to underexposure, or toxicity. Insufficient exposure to MER and PIP has been observed in adults (24, 25), and to PIP in children (26). Underexposure to antibiotics may lead to therapeutic failure and the emergence of resistant germ strains (21, 27).

We have been developing TDM of large-spectrum antibiotics in our hospital for almost two decades (28). While these efforts initially aimed at a better dosage adjustment in renal failure and renal replacement therapy (29), our attention was drawn to the issue of antibiotic underexposure, particularly in children with cancer, as pediatric oncologists gradually implemented TDM of broad-spectrum antibiotics in situations of febrile neutropenia. In this population, we aim at maintaining plasma concentrations above targets at trough.

This retrospective study evaluates, based on trough levels 1) the frequency of underexposure, 2) its association with ARC, and 3) its relation to prescribed dosages of MER or PIP in children admitted for hematologic or solid malignancies.

## Materials and Methods

### Population

This retrospective study included an unselected cohort of children aged 6 months to 18 years, admitted to the University Hospital Center of Lausanne, Switzerland for hematological or solid malignancies and in whom at least one blood level of MER or PIP was measured. The study period ran from January 2012 to end of June 2018.

### Data

Clinical data including age, gender, body weight, height, serum creatinine levels, type of malignancy, clinical indication for antimicrobial therapy, neutrophil count, together with MER and PIP dosage and sampling details were required to perform TDM interpretations and suggest dose adaptations in our routine. Our patients' data were fully anonymized before being transferred to the investigator in charge of their analysis for our research question. As patients could receive several treatment cycles, a new cycle was defined as a beta-lactam prescription initiated earliest 2 weeks after any previous beta-lactam administration. Reference MIC values for common bacteria involved in neutropenia-related infections were deduced from 90% epidemiological cut-off value (ECOFF90) from the EUCAST website (30).

### Assessment of Renal Function, Immune Status, and Infection Status

We assessed renal functions using the original Schwartz formula, which estimates creatinine clearance from serum creatinine levels in children (31). We defined ARC as a creatinine clearance value exceeding 160 mL/min/1.73 m^2^ (23, 32), and renal insufficiency as a clearance below 80 mL/min/1.73 m^2^ (33), and we assumed normal renal function between those limits. These limits are the most conservative ones among various thresholds proposed in the literature to isolate children with a well-established renal insufficiency (low limit) or a well-established ARC (high limit).

Children were classified as neutropenic if their neutrophil count was below 0.5 G/L. A bacterial infection was retained based on clinical diagnosis as listed in the medical chart, with or without an identified bacterial strain, and after having ruled out contamination.

We distinguished plasma samples taken from neutropenic febrile children treated empirically from those taken from neutropenic children with a bacterial infection, and from those sampled from immunocompetent children with bacterial infection.

### Therapeutic Drug Monitoring

All MER and PIP plasma concentrations were measured using a validated liquid chromatography assay with mass spectrometry detection (28). Separation was done using a mix of 2 solvents, ammonium formate with formic acid, and acetonitrile. This method is sensitive (limits of quantification 0.05–100 mg/L and 0.08–160 mg/L), accurate (intra/inter-assay bias 3.2/8.6 and 2.8/5.8%) and precise (intra/inter- assay CVs 6.4/3.4 and 11.3/1.9%) for MER and PIP, respectively.

TDM was not practiced systematically according to predefined schedules, but was rather done at the physician's discretion. In intermittent infusions, only blood samples drawn prior to the next dose (troughs) were included in this study, allowing however for an imprecision of ±1 h in sampling time.

In standard intermittent short infusions, the targeted concentration range at trough is between 2 and 8 mg/L for MER, and between 8 and 30 mg/L for PIP. When PIP is given as a continuous infusion, targeted levels are between 30 and 60 mg/L. While the lower limits are the efficacy thresholds given by protein-corrected MIC values of common bacteria, the upper limits are empirical and poorly defined (no established concentration-toxicity relationships). When a bacterial strain was identified in a patient, the lower targeted concentration was defined as its MIC value if available, otherwise as its bacteria-specific ECOFF90 level. These values were corrected for protein binding, although relatively low for these 2 beta-lactams (2% for MER and 15% for PIP), using the formula:

Trough plasma concentration target = (ECOFF90 or MIC) × [100/(100—percent protein binding of the beta-lactam)].

### Statistical Analyses

We evaluated the significance of comparisons using two-tailed Student's *t*-tests performed on log-transformed MER and PIP concentration values, and on native biological values otherwise. Associations between study variables were assessed by calculating log-linear or linear correlations, respectively. Imbalance between patient subgroups was evaluated with exact Fisher's test.

## Results

### Patients' and Treatment Characteristics

Altogether, 32 children provided 51 trough samples for MER and 63 for PIP, administered as intermittent short infusions (0.5 h) three, respectively four times a day during, respectively, 25 and 23 treatment cycles. Two MER and 4 PIP plasma levels were excluded because of sampling at distance from trough, and 1 PIP level as obviously aberrant. All other levels were included, tolerating a slight time deviation for 6 MER (12%) plasma levels (drawn maximum 1 h before through time), and for 15 (24%) and 12 (19%) PIP plasma levels drawn, respectively, slightly before and after trough time (maximum 1 h). In addition, continuous PIP infusions were given in three children previously on intermittent PIP administrations, which provided 13 plasma levels over six treatment cycles. Five children received both MER and PIP in different treatment cycles. The childrens' and plasma levels' characteristics are summarized in Table 1.

**Table 1:** Children and plasma levels characteristics.

**Meropenem**					**Piperacillin**		
**Population**							
19 children: 11 females/8 males					18 children: 5 females/13 males		
**Cancer**							
Hematological: 6 ALL 3 AML 1 Burkitt's lymphoma 1 Diffuse large B-cell lymphoma 1 Mature B-cell lymphoma Solid: 2 Neuroblastoma 1 Adrenocortical carcinoma 1 Medulloblastoma 1 Thoracic neuroblastoma 1 Osteosarcoma 1 Ewing sarcoma					Hematological: 6 ALL 4 AML 2 Burkitt's lymphoma 1 Mature B-cell lymphoma Solid: 2 Neuroblastoma 1 Rhabdomyosarcoma 1 Malignant rhabdoid tumor 1 Atypical teratoid rhabdoid tumor		
**Trough plasma levels under intermittent infusions**							
**51 plasma levels from 25 treatment cycles under mean dosage of 104 ± 42 mg/kg/d (min 40/max 200)**				**63 plasma levels from 23 treatment cycles under mean dosage of 336 ± 127 mg/kg/d (min 94/max 625)**			
**Immune status**	**Infection status**	**Estimated kidney function status**	**Total**	**Immune status**	**Infection status**	**Estimated kidney function status**	**Total**
Neutropenic	Bacterial infection	ARC	7	Neutropenic	Bacterial infection	ARC	10
		Normal	0			Normal	0
		Decreased	0			Decreased	1
	Febrile	ARC	8		Febrile	ARC	25
		Normal	11			Normal	10
		Decreased	4			Decreased	1
Immuno-competent	Bacterial infection	ARC	5	Immuno-competent	Bacterial infection	ARC	7
		Normal	3			Normal	1
		Decreased	0			Decreased	1
	Febrile	ARC	6		Febrile	ARC	3
		Normal	3			Normal	2
		Decreased	4			Decreased	2
**Plasma levels under continuous infusions**							
	**13 plasma levels from 3 treatment cycles under mean dosage 416 ± 104 mg/kg/d (min 132/max 541)**						
0 plasma levels	Neutropenic	Bacterial infection	ARC	3			
			Normal	1			
		Febrile	ARC	5			
	Immuno-competent	Bacterial infection	ARC	2			
			Normal	1			
		Febrile	ARC	1			

Febrile neutropenia status was highly represented, accounting for 23 (45%) of MER and 36 (57%) of PIP plasma levels measured under intermittent infusion and 6 (46%) of PIP plasma levels measured under continuous infusion. Immunocompromised children with bacterial infection were in minority, accounting for 7 (14%) of MER and 10 (16%) of PIP samples under intermittent infusion, but they were in majority for PIP samples taken under continuous infusion 7 (54%).

The daily doses given as intermittent infusions were heterogeneous, but mostly consistent with intensive dosage recommendations, i.e., 120 mg/kg/d for MER and 400 mg/kg/d for PIP (34, 35). For MER, they initially averaged 95 ± 35 mg/kg/d (mean ± SD), and increased to an average of 113 ± 49 mg/kg/d after TDM. Similarly, initial average PIP dosages were 310 ± 97 mg/kg/d with subsequent daily doses averaging 351 ± 140 mg/kg/d. Eleven MER and 17 PIP plasma levels from, respectively, 4 and 5 children were drawn under daily dosages that exceeded intensive dosage, up to 200 and 625 mg/kg/d, respectively. Continuous infusions of PIP used daily dosages of 416 ± 104 mg/kg/d.

Interestingly, initial MER prescriptions were poorly associated with the childrens' renal function (correlation coefficient R^2^ of 0.09, *p* = 0.15), with only a slight difference in average dosage between ARC status and children with normal or decreased renal function (102 ± 32 vs. 87 ± 38 mg/kg/d, *p* = 0.28). For all renal functions, the correlation improved slightly with subsequent dosage adjustments (R^2^ of 0.16, *p* = 0.04). Renal function was better taken into account in prescribing the initial dose of PIP (R^2^ of 0.14, *p* = 0.05), with higher dosages given to ARC patients (343 ± 59 vs. 238 ± 110 mg/kg/d, *p* = 0.005), and, for all renal functions, further improvement of the correlation with subsequent dosages (R^2^ of 0.31, *p* < 0.001).

### Plasma Concentrations

#### Intermittent Administration

**Figure 1:**
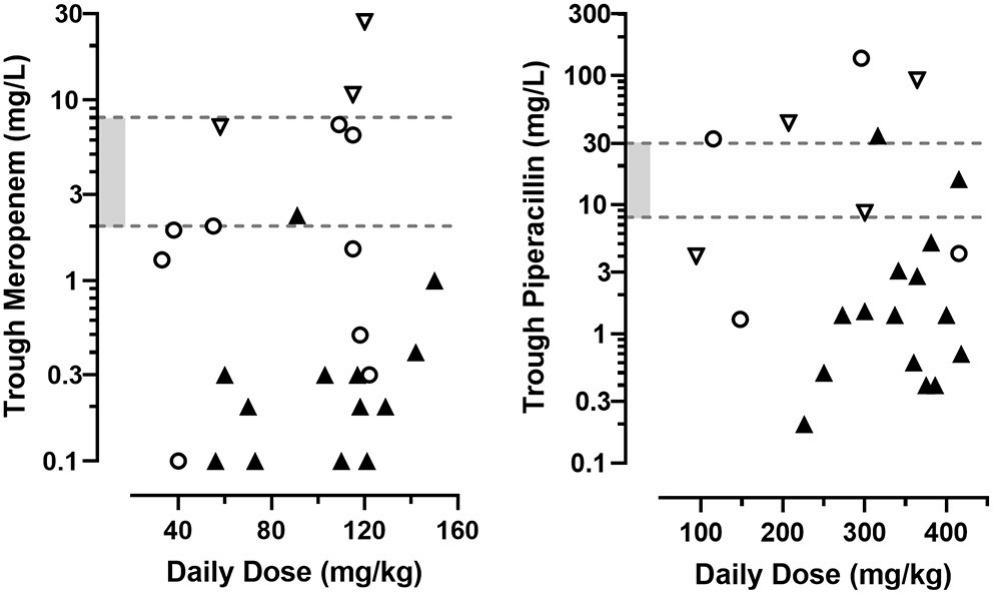
First trough concentration values of MER and PIP obtained during 25, respectively, 23 treatment cycles in a series of hospitalized pediatric hematology-oncology patients, according to the total daily dosage administered as intermittent short infusions. Shaded range is the targeted concentration interval for common bacteria. Symbols represent the patients' creatinine clearance according to the Schwartz formula: ◯ normal (80–160 mL/min/1.73 m^2^). ▽ decreased (<80 mL/min/1.73 m^2^). ▴ augmented (>160 mL/min/1.73 m^2^).

**Figure 2:**
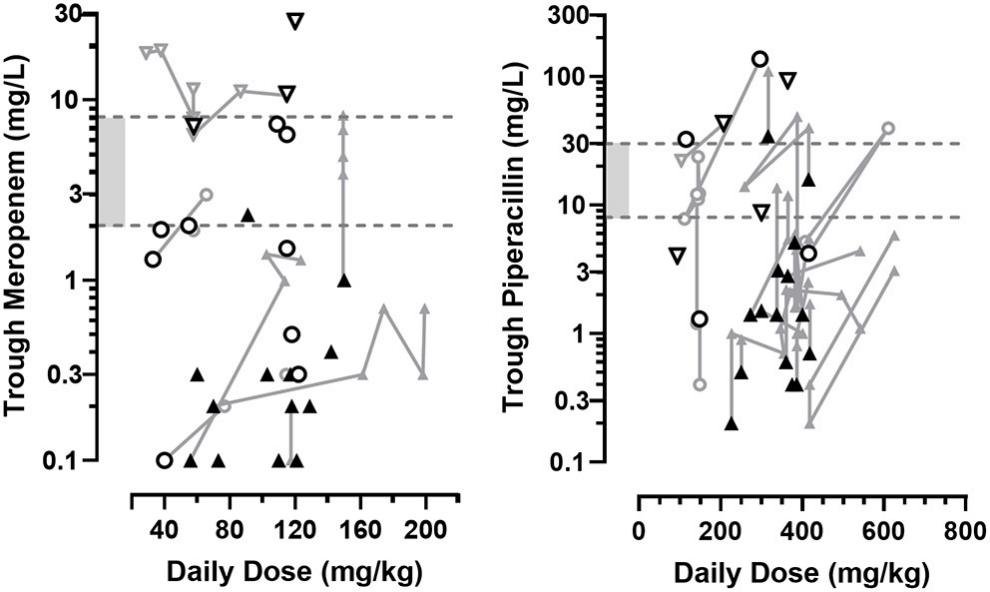
First (black) and subsequent (grey) trough concentration values of MER and PIP obtained during 25, respectively, 23 treatment cycles in a series of hospitalized pediatric hematology-oncology patients, according to the total daily dosage administered as intermittent short infusions. Measurements in individual patients are connected. Shaded range is the targeted concentration interval for common bacteria. Symbols represent the patients' creatinine clearance according to the Schwartz formula: ◯ normal (80–160 mL/min/1.73 m^2^). ▽ decreased (<80 mL/min/1.73 m^2^). ▴ augmented (>160 mL/min/1.73 m^2^).

A majority of trough plasma concentrations from patients receiving MER and PIP as intermittent infusions were below concentrations suitable for the individual situation, both at first monitored level of the treatment cycle (geometric mean ± SD, 0.7 ± 5.8 mg/L for MER and 3.4 ± 33.6 mg/L for PIP, Figure 1) as for subsequent measurements during the cycle (1.6 ± 7.1 mg/L for MER and 3.8 ± 19.8 mg/L for PIP, Figure 2). This was the case for 32 (63%) of all MER and 44 (70%) of all PIP samples. The plasma levels reached the targeted range only in 11 (21%) MER samples and 10 (16%) PIP samples. They exceeded them in 8 (16%) MER and 9 (14%) PIP samples.

When focusing on febrile neutropenic children, 13/23 (57%) and 27/36 (75%) levels of MER and PIP, respectively, were below concentrations deemed therapeutic. For children in the most critical states (i.e., with both documented bacterial infection and immunocompromised state), observed trough concentrations were below targeted concentrations in 7/7 (100%) of MER and 6/10 (60%) of PIP samples.

When pooling all individual results, trough concentrations of both MER and PIP showed no clear association with the daily doses administered, either on initial or on subsequent TDM results.

#### Piperacillin Continuous Infusion

Conversely, plasma concentrations obtained from patients receiving PIP as a continuous infusion tended to often be above the targeted concentrations, with 7 (54%) measurements exceeding 60 mg/L. The count of levels within and below the therapeutic range were 5 (38%) and 1 (8%), respectively. Most of these plasma levels were taken from neutropenic children 9 (69%) of which 4 (31%) had, in addition, a documented infection. All plasma levels of the latter patient category reached or exceed targeted concentrations (germ specific MIC or usual therapeutic range). The insufficient plasma level was observed in a neutropenic child without bacterial infection.

#### Renal Function

Most MER and PIP samples were collected from children presenting ARC, accounting for 26 (51%) of MER and 45 (71%) of PIP trough samples obtained under intermittent administrations. The renal function estimated by the Schwartz formula was deemed normal (i.e. a creatinine clearance between 80 and 160 mL/min/1.73 m^2^) for 17 (33%) MER and 13 (21%) PIP plasma levels, and decreased for the remaining 8 (16%) MER and 5 (8%) PIP measurements. Under continuous infusion, 11 (85%) PIP samples were obtained in ARC state and the rest with normal renal clearance. All plasma levels combined, nearly 2/3 of them came from children with ARC.

Trough MER concentrations measured for the first time during a treatment cycle revealed a significant inverse association with the patients' renal function (Figure 1, R^2^=0.61, *n* = 25, *p* < 0.001), which persisted on subsequent TDM determinations (Figure 2, R^2^=0.53, *n* = 26, *p* < 0.001). Accordingly, the presence of ARC was associated with significantly lower initial trough MER levels compared to levels sampled in normal and decrease renal function status (geometric mean 0.26 mg/L, *n* = 13, CV = 94% vs. 2.17 mg/L, *n* = 12, CV = 162%, *p* < 0.001), with a difference persisting in subsequent TDM measurements (0.51 mg/L, *n* = 13, CV = 143%, vs. 5.31 mg/L, *n* = 13, CV = 133%, *p* < 0.001). Similarly, higher through PIP concentrations correlated with lower renal function initially (R^2^= 0.50, *n* = 23, *p* < 0.001), but less so for subsequent measures (R^2^= 0.16, *n* = 40, *p* = 0.001), with ARC being associated with very low levels initially (1.5 mg/L, *n* = 15, CV = 141% vs. 15.5 mg/L, *n* = 8, CV = 167%). The difference in measured levels between ARC and pooled other renal functions slightly lessened in subsequent PIP TDM measurements (2.9 mg/L, *n* = 31, CV = 134% vs. 8.9 mg/L, *n* = 8, CV = 166%, *p* = 0.04).

Consequently, trough MER and PIP concentrations observed in the first TDM samples of each treatment cycles were below the recommended range in 71% of the cases. Such below target exposures were more frequent in the presence of ARC compared to normal or decreased renal function status (90% vs. 45%, *p* = 0.003). This imbalance persisted in TDM samples measured subsequently, which remained below target levels in 64% of the measurements, and most frequently observed in ARC (83% vs. 26%, *p* < 0.001). All combined, underexposure was observed much more often in samples taken from children with ARC compared to samples taken under normal or decreased renal function status (85% vs. 36%, *p* < 0.001).

## Discussion

This retrospective study emphasizes the high frequency of underexposure to broad-spectrum beta-lactam antibiotics administered as intermittent infusions in children admitted for cancer treatment, with roughly two-thirds of trough MER and PIP plasma levels found below targeted concentrations. We observed similar frequencies of low antibiotic levels in febrile neutropenic children. Underexposure was highest in the most at-risk children, i.e., those with neutropenic state and bacterial infection combined (100 and 60% for MER and PIP, respectively). This underexposure appears clearly related to the high prevalence of ARC among the young cancer patients included in this study. Acute malignancies such as leukemia or lymphoma affect children usually devoid of previous kidney impairment, allowing for significant functional reserve to be made available to recruitment in case of superimposed infection. It is only recently that this phenomenon has drawn attention as a potential source of antibiotic underexposure and therapeutic failure. Our study confirms the sizeable importance of this problem.

Our observations suggest that the dosages prescribed in our cohort were inadequate, despite most often nearing the maximum recommended daily doses (i.e., 120 mg/kg/d for MER and 400 mg/kg/d for PIP (34, 35). This “single size” daily dose led to overexposure in three children with renal insufficiency, but much more often to underexposure in relation with ARC, as observed in 26 children. Another interesting finding was that the current practice of TDM and dosage adjustment failed to correct the problem of antibiotic underexposure in most cases. Prescribers remain understandably shy on exceeding maximum recommended dosages, as iatrogenic complications may become difficult to defend. Only in a few patients did the worrisome clinical course lead the physician to prescribe MER or PIP in doses beyond those recommended, reaching 200 and 625 mg/kg/d, respectively. Use of such dosages in children have not yet been reported in the literature and no dose-dependent toxicity has been described to date for MER, while use of PIP above 600 mg/kg/d has been associated with side effects such as bone marrow toxicity (36). Still, we infer that daily doses exceeding the maximum recommendations would have little chance of causing toxicity in patients with ARC, as systemic concentrations are not expected to exceed the targeted range. We would highly encourage a change in usual practice aimed at adapting MER and PIP infusion modalities to reach the suitable therapeutic targets. As beta-lactams are time-dependent antibiotics, increased administration frequency and infusion duration will increase trough levels while foreseeably respecting the manufacturer's maximum dose. In addition, the physicochemical stability of PIP allows administration as continuous infusion. Our observations confirm that continuous infusions maintain circulating concentrations in and above the target range in most cases (92%). In contrast, MER demonstrates less stability, making multiple sequential infusions necessary for safe and effective exposure (37). Yet extended-time infusions may not be sufficient to guarantee appropriate exposure in all cases (38). An advantage of TDM in continuous infusions is that samples can be drawn at any time after reaching steady state.

Our results also raise concern that underexposure will occur with other drugs eliminated by renal filtration in young cancer patients observed to frequently present ARC. Among these is probably vancomycin (39), as shown in a study conducted in neutropenic children (32). This is of particular concern as most documented bacterial infections in cancer children are due to Gram-positive bacteria, notably *Staphylococcus aureus*.

The impact of antibiotic underexposure on clinical outcomes was difficult to assess and beyond the scope of this study. Febrile state or infection will usually delay further anticancer treatments, and recovery from chemotherapy-induced neutropenia or agranulocytosis will vary and likely contribute in helping to eradicate an infection differently in each child. In addition, data on concomitant anti-infectious agents (e.g., vancomycin, antifungals, antiviral agents) were not systematically gathered in this study.

The limitations of our study are its retrospective nature and a rather limited number of samples. Yet we did not identify any previous study having assessed MER and PIP exposures in cancer children clearly taking the impact of ARC into account (26, 40). This could be explained by the restricted hematology-oncology patient population and the still restricted availability or adopted practice of TDM for beta-lactams.

Applying TDM in this clinical setting was not standardized, as only a prospective design could have guaranteed systematic measurements in children receiving MER or PIP following cancer therapy. A selection bias may thus in part explain the very high frequency of ARC among our patients, exceeding the 30% to 40% reported in previous publications for this population (22, 23). TDM may have been preferably performed in patients with unsatisfactory outcome, and children with ARC resulting in underexposure could be overrepresented. Even if ARC is rather common in children experiencing various pathological states, it seems to occur with a highly variable frequency (41, 42).

Another limitation is some weakness in estimating the renal function, inherent to the Schwartz's formula (43). Serum creatinine values may not always appropriately reflect muscle mass, especially after prolonged hospitalization or in neurological diseases. Moreover, this formula was developed using measurements from children with impaired or normal renal function. Its performance may be questioned in the higher range of clearance values that characterize ARC (31). Therefore, we opted for a high cut-off at 160 mL/min/1.73 m^2^ to define ARC and minimize the risk of misclassifying patients.

The reliability of blood sampling information or its transcription could also be a limitation. In intermittent infusions, included samples were presumably drawn at time of trough ±1 h. Nonetheless, the very short half-lives of MER and PIP (~1 and 0.7 h, respectively) easily makes sampling inaccuracy translate into a significant imprecision in concentration result. As more samples were taken rather slightly earlier than later to the trough time, blood levels would more often have been overestimated.

Our definition of target ranges for MER and PIP trough concentrations might be disputed. While most authors agree on recommending to maintain a protein-corrected MIC coverage throughout the entire dosing interval in neutropenic and critically ill patients (44, 45), lower targets may be sufficient in immunocompetent children. A minimum of 40 to 50% of the dosing interval time above MIC is usually tolerated to ensure a bactericidal effect of MER or PIP in immunocompetent patients (46, 47).

In conclusion, the administration of MER and PIP as intermittent infusions often fails to ensure sufficient concentration exposure in children admitted for cancer treatment developing febrile neutropenia. This mainly results from a state of ARC, frequently observed during acute malignancies and episodes of febrile neutropenia. First steps to ensure proper antibiotic coverage in these patients will probably require modifying treatment modalities (prolonged infusion time, continuous infusion for PIP) and eventually increasing daily dosages beyond those currently recommended. Thorough assessment of renal function, possibly with improved diagnostic tests, and systematic TDM seem clearly advisable in such conditions. Prospective clinical trial remain warranted to validate the benefits and the safety of such suggested practice changes.

## Data Availability Statement

The raw data supporting the conclusions of this article will be made available by the authors, without undue reservation.

## Ethics Statement

Ethical review and approval was not required for the study on human participants in accordance with the local legislation and institutional requirements. Written informed consent from the participants' legal guardian/next of kin was not required to participate in this study in accordance with the national legislation and the institutional requirements.

## Author Contributions

PA, LD, and KD contributed to conception and design of the study. PA organized the database. TB analyzed data and performed the statistical analysis. PA and LR wrote the first draft of the manuscript. PC, HC, LD, MD, and SA wrote sections of the manuscript. All authors contributed to manuscript revision, read, and approved the submitted version.

## Conflict of Interest

The authors declare that the research was conducted in the absence of any commercial or financial relationships that could be construed as a potential conflict of interest.

## Publisher's Note

All claims expressed in this article are solely those of the authors and do not necessarily represent those of their affiliated organizations, or those of the publisher, the editors and the reviewers. Any product that may be evaluated in this article, or claim that may be made by its manufacturer, is not guaranteed or endorsed by the publisher.

## Abbreviations

ALL: Acute lymphoblastic leukemia
AML: Acute myeloblastic leukemia
ARC: Augmented renal clearance
ECOFF90: Epidemiological cut-off values covering 90% of germ strains
MER: Meropenem
MIC: Minimal inhibitory concentration
PIP: Piperacillin
TDM: Therapeutic drug monitoring.
